# Formulation, Characterization and Biological Activity Screening of Sodium Alginate-Gum Arabic Nanoparticles Loaded with Curcumin

**DOI:** 10.3390/molecules25092244

**Published:** 2020-05-10

**Authors:** Abdelkader Hassani, Syed Mahmood, Hamid Hammad Enezei, Siti Aslina Hussain, Hamad Ali Hamad, Ahmed Faris Aldoghachi, Abdullah Hagar, Abd Almonem Doolaanea, Wisam Nabeel Ibrahim

**Affiliations:** 1 Department of Pharmaceutical Technology, Faculty of Pharmacy, International Islamic University Malaysia, Kuantan 25200, Malaysia; hassani.abk@gmail.com; 2 Department of Chemical and Environmental Engineering, Faculty of Engineering, University Putra Malaysia, UPM, Serdang 43400, Malaysia; aslina@upm.edu.my; 3 Department of Pharmaceutical Engineering, Faculty of Chemical and Process Engineering Technology, University Malaysia Pahang, Gambang 26300, Malaysia; syedmahmood@ump.edu.my; 4 Centre of Excellence for Advanced Research in Fluid Flow (CARIFF), University Malaysian Pahang, Gambang 26300, Malaysia; 5 Department of Oral and Maxillofacial Surgery, Collage of Dentistry, University of Anbar, Ramadi, Iraq; drhamed2000@yahoo.com; 6 Department of Biomedical Sciences, Faculty of Medicine and Health Sciences, University Putra Malaysia, UPM, Serdang 43300, Malaysia; hamadali91.ha@gmail.com (H.A.H.); ahmed.faris2012@hotmail.com (A.F.A.); 7 Department of Nutrition Sciences, Faculty of Allied Health Sciences, International Islamic University Malaysia, Kuantan 25200, Malaysia; hajar@iium.edu.my; 8 Department of Biomedical sciences, College of Health sciences, QU Health, Qatar University, Doha, Qatar

**Keywords:** curcumin, gum Arabic, sodium alginate, nanoparticles, cytotoxicity

## Abstract

The approach of drug delivery systems emphasizes the use of nanoparticles as a vehicle, offering the optional property of delivering drugs as a single dose rather than in multiple doses. The current study aims to improve antioxidant and drug release properties of curcumin loaded gum Arabic-sodium alginate nanoparticles (Cur/ALG-GANPs). The Cur/ALG-GANPs were prepared using the ionotropic gelation technique and further subjected to physico-chemical characterization using attenuated total reflectance–Fourier transform infrared (ATR-FTIR), X-ray diffractometry (XRD), differential scanning calorimetry (DSC), size distribution, and transmission electron microscopy (TEM). The size of Cur/ALG-GANPs ranged between 10 ± 0.3 nm and 190 ± 0.1 nm and the zeta potential was –15 ± 0.2 mV. The antioxidant study of Cur/ALG-GANPs exhibited effective radical scavenging capacity for 1,1-diphenyl-2-picrylhydrazyl (DPPH) at concentrations that ranged between 30 and 500µg/mL. Cytotoxicity was performed using MTT assay to measure their potential in inhibiting the cell growth and the result demonstrated a significant anticancer activity of Cur/ALG-GANPs against human liver cancer cells (HepG2) than in colon cancer (HT29), lung cancer (A549) and breast cancer (MCF7) cells. Thus, this study indicates that Cur/ALG-GANPs have promising anticancer properties that might aid in future cancer therapy.

## 1. Introduction

Drug development for the treatment of cancer is currently one of the most extensive areas being funded and researched. However, among the many obstacles facing these therapeutic agents is their limited absorption and retention by cancer cells which also lead to toxic effects in normal tissue cells [1,2,3,4]. In this quest, the development of nanoparticle formulations of different therapeutic agents served in providing a better delivery tool of chemotherapies with better dosage and targeting precision to the tumor site [5,6,7,8,9].

Among the chemotherapeutic agents in cancer, there is a growing interest in natural products for their effectiveness and lower toxicity [10]. Nanoparticles formulated with the chemotherapeutic hydrophobic agents like curcumin have shown promising outcomes in the treatment of solid tumors such as breast cancer, liver cancer, and pancreatic cancer [11,12,13,14,15]. Curcumin, a yellow hydrophobic polyphenol obtained from the turmeric’s rhizome with the chemical composition of diaryheptanoid bis-α,β-unsaturated β-diketone known as diferuloylmethane [16,17,18]. Curcumin possesses diverse therapeutic applications in chronic inflammatory diseases such as cardiovascular diseases and in cancer cells due to its antioxidant and pro apoptotic properties [19]. However, these medical applications were challenged by the poor bioavailability of Curcumin at the target tissue site. The reduction in curcumin’s bioavailability mainly results from the poor solubility, the rapid elimination, extensive intestinal, and hepatic metabolism, and its accelerated degradation at physiological pH [20]. Several approaches have been taken to address these obstacles by including techniques applicable to the administration and encapsulation of curcumin using biodegradable polymers. Using these polymers provides a feasible approach to enhance the bioavailability of curcumin [21].

The antioxidant capacity in the assessment of nanoparticles potential is the most studied property among the therapeutic properties in nanomedicine and pharmaceutical sciences. The free radicals generated from the interaction between biomolecules and molecular oxygen cause the degradation of biomolecules in the biological system. Many types of natural antioxidants play a crucial role in the scavenging of toxic free radicals and inhibition of oxidation reactions [22]. As mentioned by Chen et al. the antioxidant capacity toward DPPH is significantly enhanced by nanoencapsulation. In addition, curcumin is known to have a potent free radicals scavenging activity due to the capability of curcumin to reduce DPPH radicals based on the denotation of an H-atom from its central heptadienone linkage where the nanostructured form is expected to react as better alternative for free radical scavengers due to the high surface volume ratio of curcumin nanoformulations [23].

Sodium alginate is a salt derivative of alginic acid, a natural polysaccharide polymer of M residues (1,4-linked β-D-mannuronic acid) and G residues (1,4 α-L-guluronic acid), derived from brown algae cell walls, including *Macrocystis pyrifera*, *Laminaria hyperborea*, *Ascophyllum nodosum* [24]. As an anionic polysaccharide, sodium alginate is widely used because of its non-toxicity, biocompatibility and its physical transition state due to its crosslinking properties. The nanoformulation using sodium alginate is a valuable tool for the encapsulation of bioactive agents that serves as a functional system in the pharmaceutical applications. Studies using sodium alginate to load therapeutic agents such as doxorubicin or paclitaxel has shown an improvement in the selectivity of drug release in cancer cells with improving safety profile [25,26]. In addition, the preparation of silver nanoparticles by sodium alginate as a stabilizer has shown potential antibacterial activity via in vitro assays [27].

Gum Arabic (GA) is also a natural composite, a highly water-soluble polysaccharide containing galactose and arabinose, also known as acacia gum, frequently extracted from exudates of *Acacia Senegal*. In the last decades, GA has been used widely as an additive ingredient due to its antioxidant activity, low viscosity at high temperature, and binding properties [28]. The use of GA as a coating material for therapeutic agents included formulated gold nanoparticles which demonstrated potent antioxidant properties in addition to other therapeutic properties in cancer cells using mice models of lung cancer, leading to the conception of recommending its role in the preparation of nanoparticles for further diagnostic and therapeutic uses [29,30].

In same context, GA was used to overcome the limitation of selenium toxicity and improved its delivery and stability for anticancer properties [31].

Giving the information above, it was decided to enhance the anticancer activity of curcumin in a novel polymer nanoparticles formulation using gum arabic (GA) and sodium alginate (ALG). The use of both polymers intended to improve the bioavailability, release, and therapeutic properties of curcumin due to the crosslinking reaction between the hydroxyl groups of both polymers. Using the ionotropic gelation method of combining the two polymers also provides an additional protective measure for drugs in the gastric tract [32,33,34].

In the current study, curcumin (Cur) encapsulated into sodium alginate- gum Arabic nanoparticles was characterized, and the antioxidant activity against DPPH was assessed with different time intervals and concentrations of nanoparticles. This is the first time reporting the effectiveness of using sodium alginate/gum arabic-curcumin nanoparticles as an improved delivery tool of curcumin in number of cancer cell lines including MCF7, HepG2, HT29, and A549 cancer cells demonstrating an increased antioxidant and anti-proliferative properties. Therefore, this study supports the use of both polymers in nanomedicine applications.

## 2. Results

**Figure 1:**
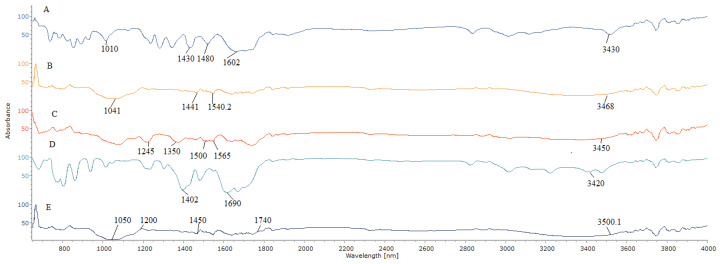
ATR-FTIR spectra of (**A**) ALG, (**B**) GA, (**C**) Free curcumin, (**D**) ALG-GA nanoparticles, (**E**) Cur/ALG-GANPs.

Attenuated total reflectance-Fourier transform infrared (ATR-FTIR) was used to provide additional information about the interactions inside the nanoparticles systems. Figure 1 shows the ATR-FTIR spectra of the ALG, GA, free curcumin (Cur), ALG-GA nanoparticles, and Cur/ALG-GANPs. The band in ALG spectrum at 3430 cm^−1^ was attributed to hydroxyl groups, while the bands at 1602 cm^−1^ and 1430 cm^−1^ were due to the symmetric and asymmetric stretching vibrations of the groups COO^−^, respectively. The band recorded at 1010 cm^−1^ was due to the saccharide structure (C-O-C). The spectrum of GA with band characteristics is shown in Figure 1B. The band at 3468 cm^−1^ was attributed to the O-H stretching and C-O bending was present at 1441 cm^−1^. The C-H stretch vibration was present at 1441 cm^−1^ and 1041.61 cm^−1^. The ATR-FTIR spectra of curcumin revealed an absorption band at 3450 cm^−1^ assigned to the phenol O-H stretching vibration. In addition, the sharp peaks at 1565 cm^−1^ and 1500 cm^−1^ were attributed to the stretching vibration of the benzene ring and the vibrations of (C-C, C=O), respectively. The characteristic peaks observed at 1305 cm^−1^ and 1240 cm^−1^, were associated with the C-H olefinic bending and the C-C aromatic stretching vibrations, respectively. For ALG-GA nanoparticles (Figure 1D), the characteristic peaks at 3420 cm^−1^ and 1690 cm^−1^ were related to the hydroxyl groups of ALG polymer and the vibrations of the function COO^−^, respectively. The C-O bending of gum arabic was assigned at 1402 cm^−1^, indicating the presence of both polymers in ALG-GA nanoparticles.

Meanwhile, the band of Cur/ALG-GANPs (Figure 1E) can be seen at 3500.1 cm^−1^ (phenolic –OH group), and a peak at 1740 cm^−1^ (C-C, C=O). The absorption bands of 1602 cm^−1^ and 1480 cm^−1^ (Figure 1A) were shifted to 1740 cm^−1^ and 1450 cm^−1^ (Figure 1E), respectively, after the complexation with gum arabic (GA). From the results obtained (Figure 1B), the peaks of 1441 cm^−1^ and 1041 cm^−1^ were shifted also after the complexation of GA with ALG to 1450 cm^−1^ and 1200 cm^−1^, respectively.

**Figure 2:**
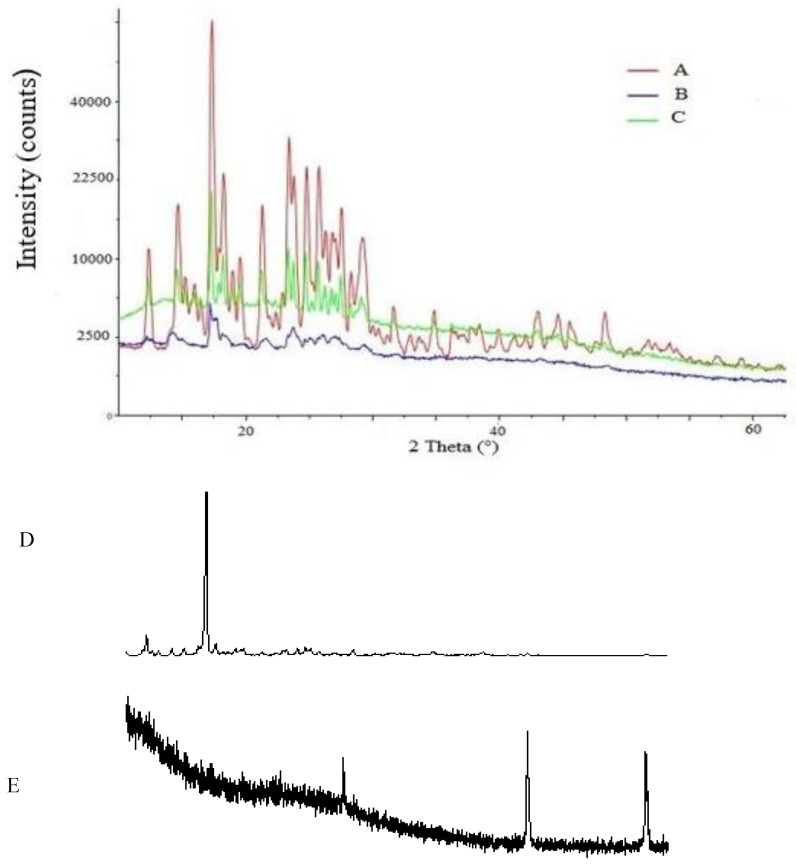
XRD analysis of (**A**) Free curcumin, (**B**) ALG-GA nanoparticles, (**C**) Cur/ALG-GANPs (**D**) Alginate, (**E**) Gum arabic.

The X-ray diffraction analysis was carried out to examine the crystallinity of curcumin after being entrapped into ALG-GA nanoparticles. The pattern of curcumin consists of sharp crystalline peaks at 15°, 17.2°, 18°, 23°, 23.7°, 24.9°, 26.9°, 29.12°, and 32° as shown in Figure 2. In the X-ray pattern of ALG, the shape peaks were revealed at several diffraction angles 16.22°, 17.94°, and 22.11° which indicated the crystalline phase of polymer (Figure 2D). On the other hand, there was 2 sharp peaks of the pattern of GA, at 27.3° and 42.5° (Figure 2E). X-ray diffraction patterns of Cur/ALG-GANPs exhibited a sharp peaks at 5°, 10.2°, 16.3°, 17.92°, 20.1°, 23.1°, 25.17°,27°, and 29°. The ALG-GA nanoparticles revealed sharp peaks at 5.03°, 13.11°, 16.21°, 20.14°, 23.64°, 25.16°, and 28.90° (Figure 2C). Some of ALG, GA, and curcumin peaks were vanished due to the complexation that happened between ALG-GA and curcumin.

**Figure 3:**
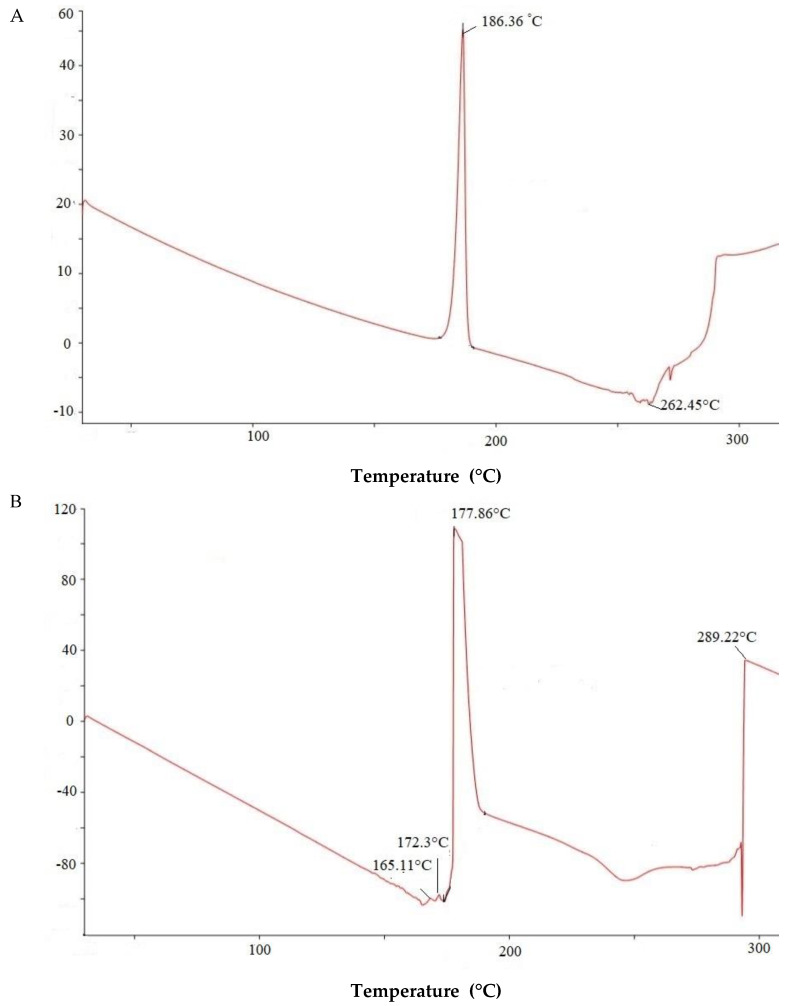

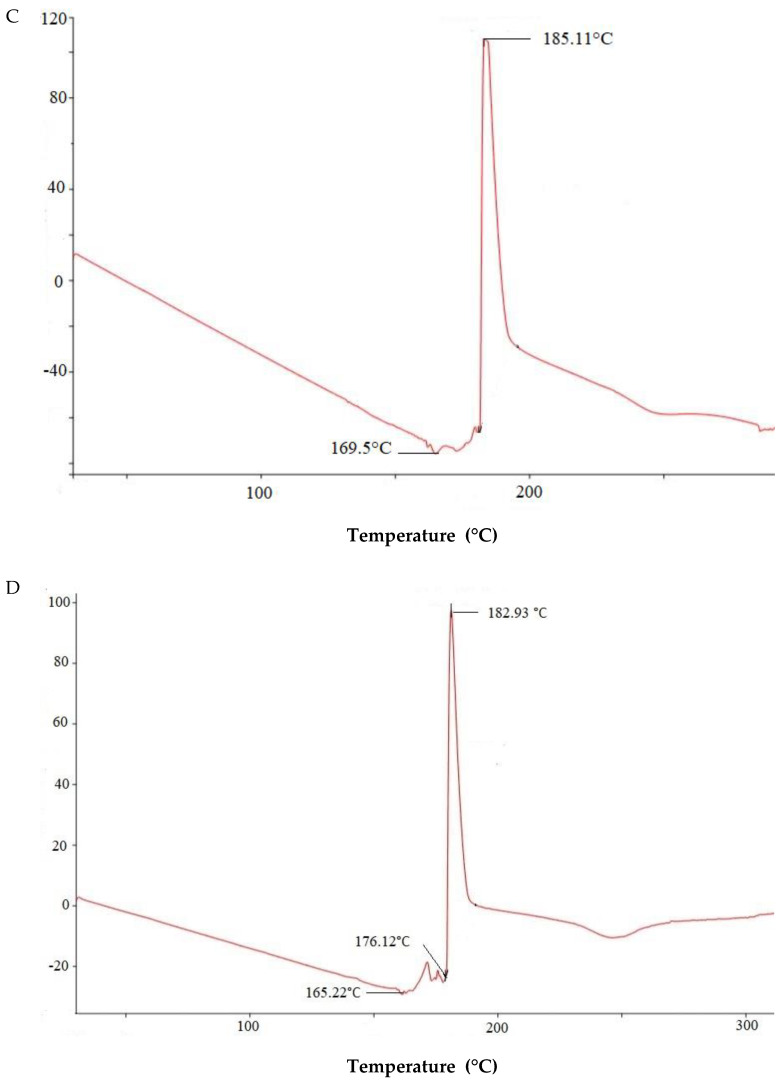

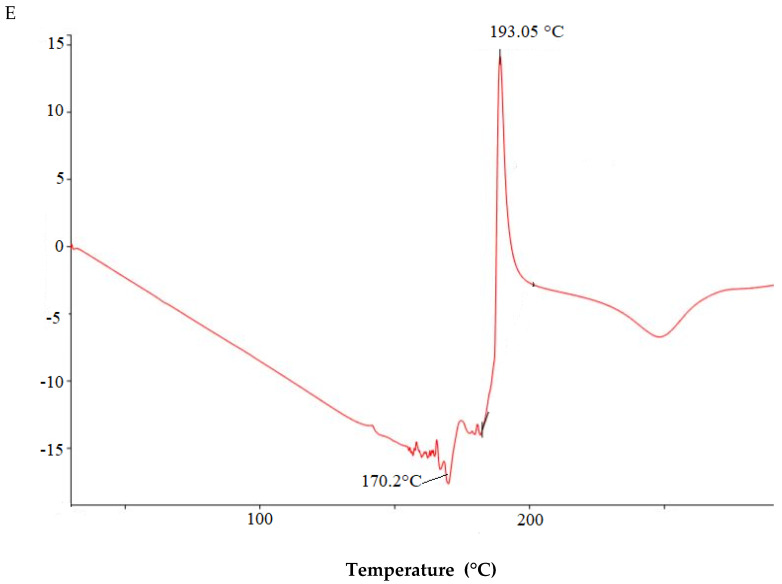
DSC curves of (**A**) Free curcumin, (**B**) GA polymer, (**C**) ALG polymer, (**D**) ALG-GA nanoparticles, (**E**) Cur/ALG-GANPs.

The DSC analysis was performed to record the thermal behavior and crystallinity of the formulation. DSC thermograms of Cur/ALG-GANPs and those for Curcumin, ALG, GA, ALG-GA nanoparticles, are depicted in Figure 3. For curcumin pattern (Figure 3A), two main thermal events were clearly indicated. The first one is an endothermic peak at 186.36 °C which is attributed to the melting point of curcumin. The second stage at 262.45 °C is due subtle decomposition and combustion of curcumin.

The DSC thermogram of GA (Figure 3B) demonstrated a first region at 165.11–177.86 °C, attributable to melting of gum arabic. The second region at 289.22 °C was due to the decomposition of gum arabic.

DSC thermogram of sodium alginate (Figure 3C) exhibited an endothermic peak at 185.11 °C corresponding to melting of alginate. The exothermic peak at 169.5 °C was correlated to the loss of water related to the hydrophilic functions of ALG polymer. The thermal decomposition of ALG-GA nanoparticles (Figure 3D) occurred at temperature maxima of 165.22 °C and 176.12 °C. The peak at 182.93 °C most probably reflects a combination of the endothermic peaks of two polymers.

The first weight loss of Cur/ALG-GANPs (Figure 3E) was carried out at 170.2 °C. The second stage is overlapped and can be attributed to the decomposition of ALG-GA nanoparticles and the combustion of curcumin at 193.05 °C.

**Figure 4:**
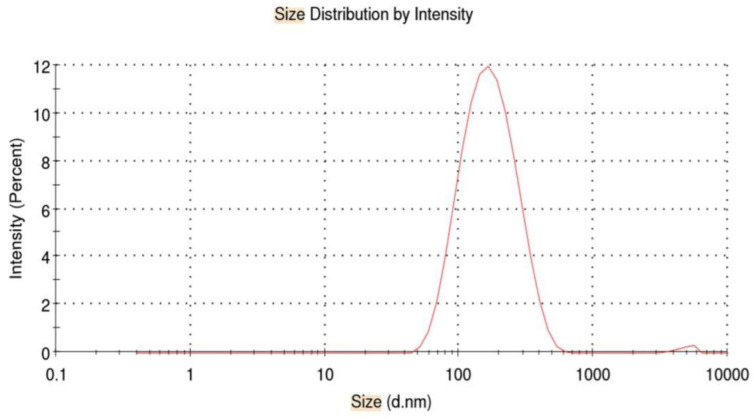
Size distribution of Cur/ALG-GA NPs.

**Figure 5:**
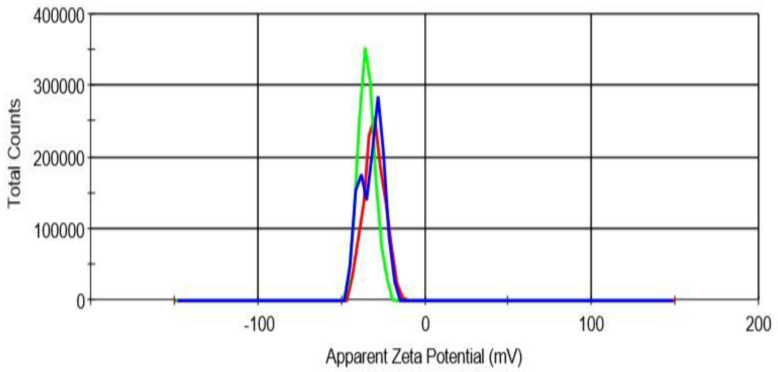
Zeta potential of Cur/ALG-GA NPs.

As depicted in Table 1, the particle size of Cur/ALG-GANPs nanoparticles ranged from 10 ± 0.3 to 190 ± 0.11 nm, while the zeta potential was −15 mV ± 0.22 (Figure 4; Figure 5).

**Figure 6:**
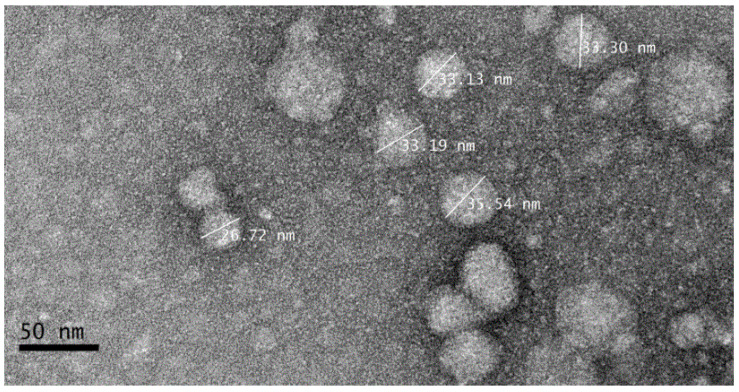
Transmission electron microscopy of Cur/ALG-GA NPs.

The morphology and extent dispersion of nanoparticles were carried out at room temperature using transmission electron microscopy (TEM). TEM image analysis of Cur/ALG-GANPs nanoparticles are depicted in Figure 6. It is apparent that Cur/ALG-GANPs are approximately round and spherical in shape with diameter ranging from 33 nm to 72 nm.

The encapsulation efficiency of curcumin within the Cur/ALG-GANPs nanoparticles was found to be > 89 ± 0.2% based on the calculations explained in the methods. The high ratio of entrapment might be due to the emulsification and gelling properties of gum arabic (27).

**Figure 7:**
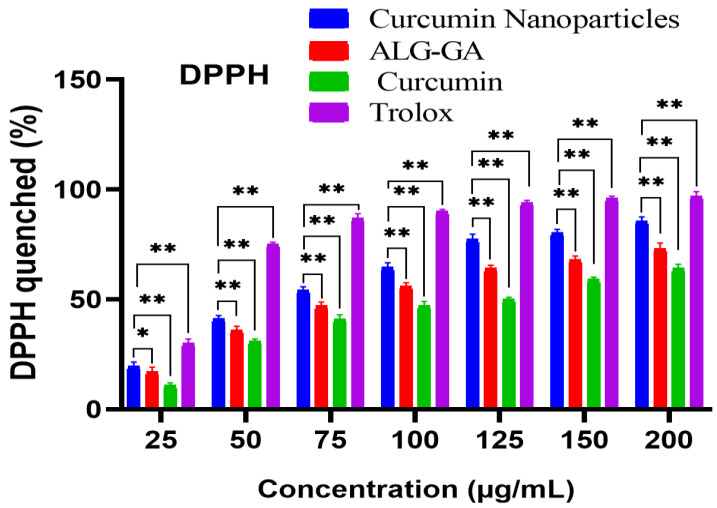
The DPPH scavenging activities at concentrations that affect ALG-GA nanoparticles, curcumin loaded in ALG-GA NPs, and Curcumin alone. Mean ± SD of three experiments. compared to Trolox used as a positive controls. Error bar are * *p* < 0.05, ** *p* < 0.01.

The antioxidant activities of curcumin loaded nanoparticle (Cur/ALG-GANPs), ALG-GA nanoparticles, and curcumin alone compared to Trolox were assessed based on the DPPH assay due to its electron property to display a violet colour in the solution. DPPH is a compound possessing an odd electron and empty space in its orbitals as well as a strong UV/Vis absorption property at 517 nm. In this study, Trolox was used as a positive control due to its capacity to dissolve in the aqueous system [35,36,37,38]. The notable antioxidant properties in this test were elicited with concentrations of 25, 50,100, and 200 µg/mL, where the % of DPPH scavenging activities of Cur/ALG-GANPs were 19.67, 41.23, 64.5, and 85.6% respectively, while for the same concentrations of free curcumin the percentages of DPPH scavenging activity were 11.12%, 31.26%, 47.3%, and 64.23%, respectively (*p* < 0.05). The scavenging activity varied significantly among different concentrations and samples. Trolox exhibited a scavenging activity of 90% and 97.01% on DPPH at the concentrations of 100 µg/mL and 200 µg/mL, respectively, as shown in (Figure 7). At the concentration of 200 µg/mL, significant differences of 11.41% and 32.78% were recorded between Trolox and curcumin nanoparticles (Cur/ALG-GANPs) and between Trolox and curcumin solution, respectively. Concentrations below of 25 µg/mL of the curcumin nanoparticles, empty nanoparticles, curcumin, and Trolox used in the cytotoxicity study didn’t have convincing antioxidant activity in the DPPH test and were therefore omitted in the analysis. The results indicated that the potent antioxidant activity in nanoparticles loaded curcumin ranged from 64.5–85.6% over the concentrations of 100–200 µg/mL. ALG-GA nanoparticles showed more potent antioxidant capacity than curcumin and lower than Trolox and nanoparticles loaded curcumin. The differences in antioxidant activities between nanoparticles with curcumin and empty nanoparticles (ALG-GA) were 7.6 % and 12.35% at the concentrations of 100 µg/mL and 200 µg/mL, respectively. The antioxidant activities of nanoparticles loaded curcumin were slightly higher than of ALG-GA nanoparticles in all concentrations but were more significant between 100 µg/mL and 200 µg/mL. Therefore, the significant differences in scavenging activity between Trolox and nanoparticles were 33.1% and 23.76% at the concentrations of 100 µg/mL and 200 µg/mL, respectively. Hence, both sodium alginate and gum arabic improve the antioxidant properties of curcumin in a dose dependent manner.

**Figure 8:**
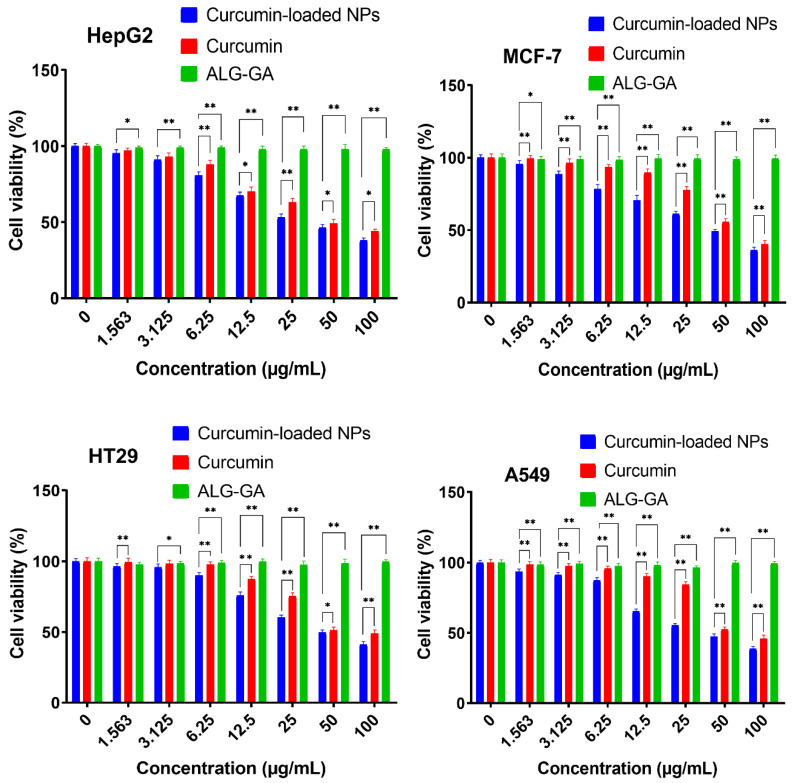
Cytotoxicity activity of Curcumin and curcumin-loaded NPs at different concentrations exposed to cancer cell lines (MCF7, HepG2, A549, and HT29) after 24 h. Mean ± SD of three experiments. Error bar are * *p* < 0.05, ** *p* < 0.01.

The cytotoxic activity of free curcumin and Cur/ALG-GANPs were measured for liver cancer (HepG2), lung cancer (A 549), colon cancer (HT29), and breast cancer (MCF7) cell lines (Figure 8). The colourimetric determination of cell viability was carried out after 24, 48, and 72 h of incubation.

The lowest cell viability was seen in HepG2 cells whereby the IC_50_ of curcumin-loaded Cur/ALG-GANPs was 9.64 ± 1.1 while that of curcumin was 19.02 ± 1.3. The IC_50_ of Cur/ALG-GANPs was found to be between 9.64 µg/mL to 16.84 µg/mL while that of free curcumin ranged from 19.02 µg/mL to 32.01 µg/mL among the four cancer cell lines (Table 2 and Table 3). These results show an almost 50% reduction of the values of IC_50_ with Cur/ALG-GANPs nanoparticles. The empty nanoparticles had no inhibitory effect on cancer cell proliferation even by increasing its concentration. The cytotoxicity assay indicated that curcumin nanoparticles were more efficient than free curcumin in inhibiting cancer cell growth.

## 3. Discussion

The peaks of ALG and GA were shifted due to the complexation of curcumin in sodium alginate-gum Arabic nanoparticles. The ATR-FTIR spectrum of Cur/ALG-GANPs also revealed the combination of both functional groups of polymers and curcumin, proving that curcumin was successfully encapsulated. In short, the Cur/ALG-GANPs had significant characters of curcumin in the ATR-FTIR spectrum, suggesting that there was no interaction between the polymers used and curcumin. Hence, the results confirmed the presence of curcumin in the ALG-GA NPs nanoparticles.

The curcumin nanoparticles did not display crystalline peaks due to the formation of amorphous state during the nanoparticle systems preparation. In Figure 2C, some of curcumin peaks were vanished and the XRD spectra showed that the characteristic peaks in X-ray diffractions for free curcumin entrapped in the XRD pattern of Cur/ALG-GANPs nanoparticles Figure 2C.

The thermogram of Cur/ALG-GANPs revealed a different peak from those of ALG-GA nanoparticles even they appeared to be an association of both polymers due to the new chemical bounds resulted from the complexation of polyelectrolyte. It could be seen that the exothermic peak of sodium ALG-GA nanoparticles appeared at 182.93 °C, a proximate intermediate peak, in comparison with the peaks of both polymers (177.86 °C and 185.11 °C) which can be explained by the interactions occurring between polyelectrolytes.

The presence of exothermic peak is due to the process of degradation of polyelectrolyte caused by the reactions of depolymerisation and dehydration which occurred most probably due to the oxidation of polyelectrolyte as well as the partial decarboxylation of protonated carboxylic groups.

The final stage of Cur/ALG-GANPs was related to the formation of Cur/ALG-GANPs phase at 193.05 °C. Increasing the decomposition of curcumin from 186.36 to 193.05 °C indicates the thermal stability of curcumin in the nanoparticle form.

The preparation of Cur/ALG-GANPs was performed using ionotropic gelation method with slight modifications. The radical scavenging activity of both free Curcumin and Cur/ALG-GANPs was carried out following the literature. This assay was focused on the evaluation of DPPH reduction in alcoholic solution. The main purpose of this test was to assess the antioxidant activity of curcumin before and after encapsulation in ALG-GANPs nanoparticles due to the sensitive, rapid, reproducible, and simple assay. The decrease of characteristic maximum absorption of DPPH at 517 nm was related to the concentration of the non-radical form of DPPH. Upon the reduction by an antioxidant, the reduction of DPPH into non-radical diphenylpicrylhydrazine was performed by the donation of the H atom of the antioxidant. The curcumin encapsulated in ALG-GA nanoparticles exhibited more DPPH scavenging activity due to its hydrogen-donating ability. The DPPH molecules were scavenged inside the nanoparticles based on the high hydrophobicity of DPPH in nature. At the same conditions, it might suggest the influence of the central methylenic hydrogen of the heptadienone moiety or the phenolic on the antioxidant activity of free curcumin. It has been reported that the keto form dominates in both neutral and acidic aqueous solutions as well as curcumin molecule reacts as an excellent potent H-atom donor. Moreover, the determination of the antiradical activity of an antioxidant was carried out by evaluating a decrease in the absorbance of DPPH• at 517 nm. A stable DPPH• radical was formed after the scavenging of DPPH• through the donation of hydrogen or electron from the antioxidant. Then, the absorbance at 517 nm was disappeared and this molecule turn into a diamagnetic stable form. The keto form of curcumin includes a high carbon activated atom between two methoxyphenol rings of the heptadienone linkage which can abstract a hydrogen atom from the carbon atom [39].

**Figure 9:**
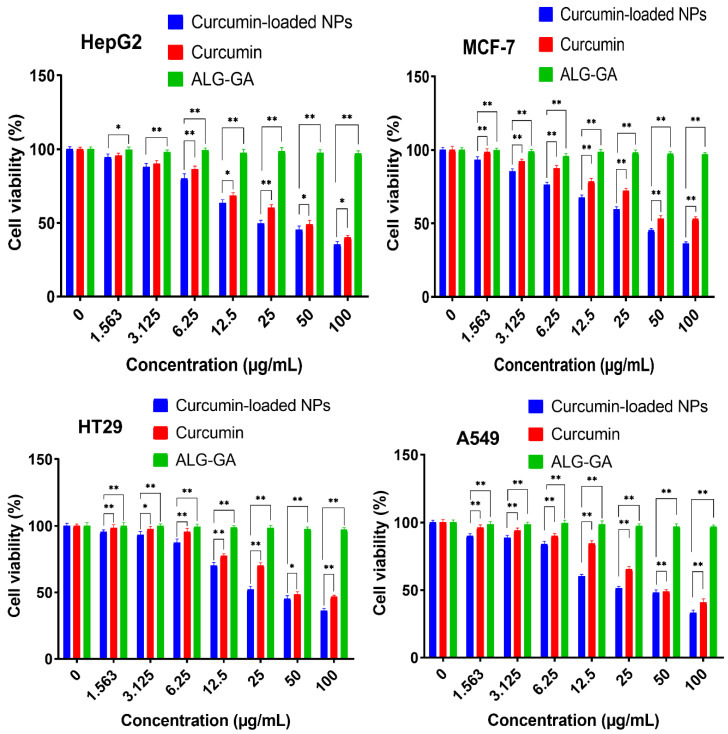
Cytotoxicity activity of Curcumin and curcumin-loaded NPs at different concentrations exposed to cancer cell lines (MCF7, HepG2, A549, and HT29) after 48 h. Mean ± SD of three experiments. Error bar are * *p* < 0.05, ** *p* < 0.01.

**Figure 10:**
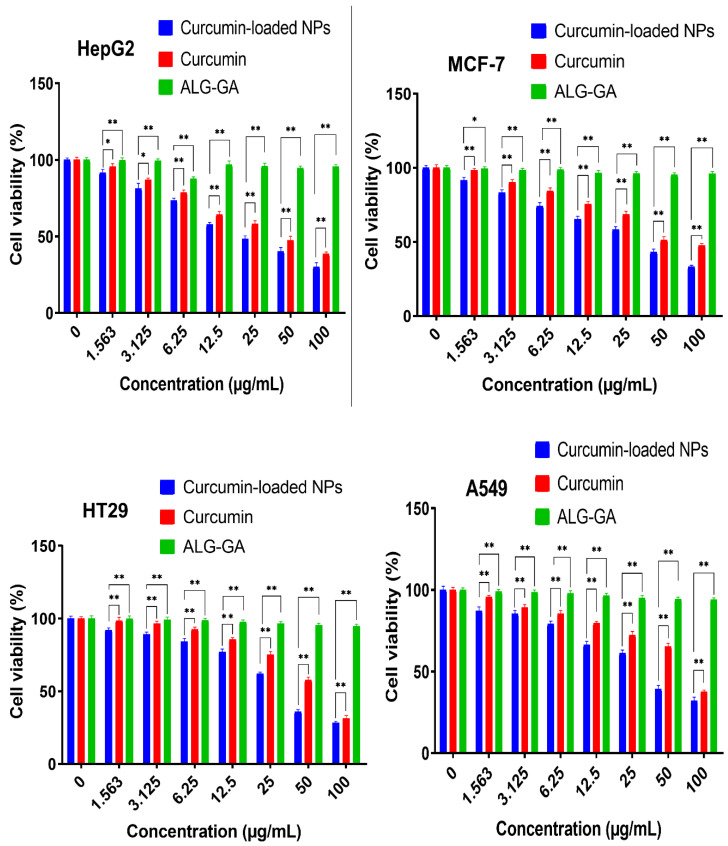
Cytotoxicity activity of Curcumin and curcumin-loaded NPs at different concentrations exposed to cancer cell lines (MCF7, HepG2, A549, and HT29) after 72 h. The values were the mean *±* SD from three independent experiments expressed as percent control. Error bar are * *p* < 0.05, ** *p* < 0.01.

**Figure 11:**
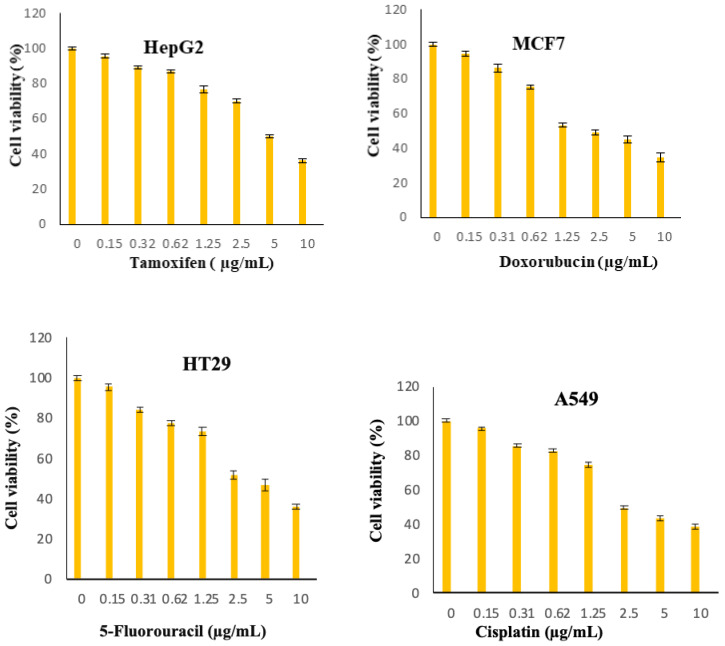
Chemotherapeutic effects on the viability of treated cells (MCF7, HepG2, A549, and HT29) using MTT assay after 24 h.

**Figure 12:**
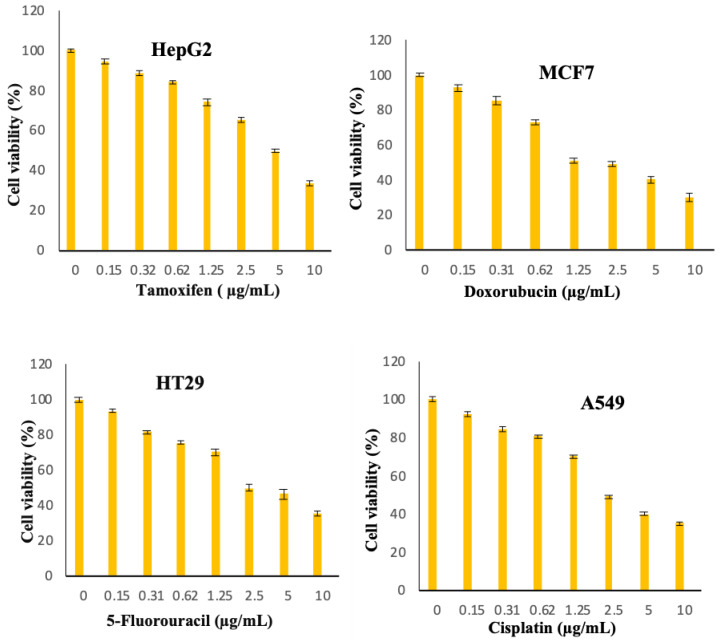
Chemotherapeutic effects on the viability of treated cells (MCF7, HepG2, A549, and HT29) using MTT assay after 48 h.

**Figure 13:**
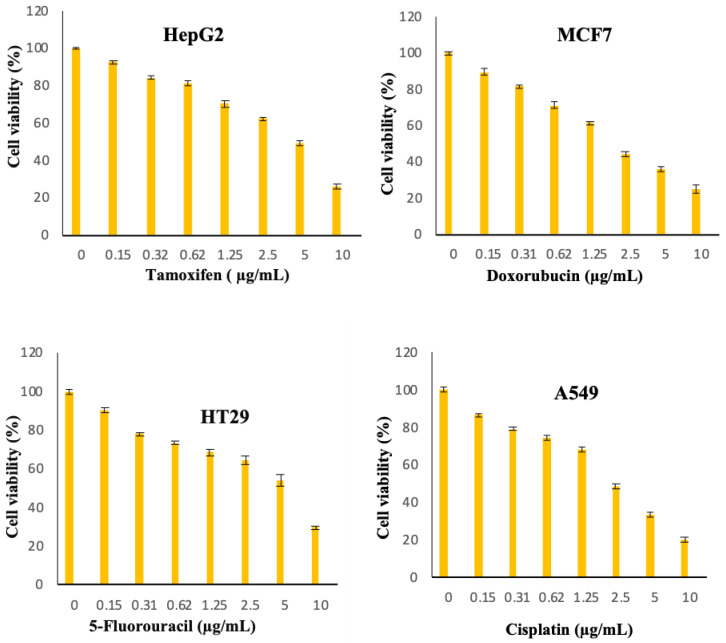
Chemotherapeutic effects on the viability of treated cells (MCF7, HepG2, A549, and HT29) using MTT assay after 72 h.

Figure 8, Figure 9, Figure 10, Figure 11, Figure 12, Figure 13 show the effect of Cur, Cur/ALG-GANPs, and chemotherapeutics towards MCF7, HepG2, HT29, and A549 cells by the MTT assay after 24, 48, and 72 h of exposure. The MTT assay is based on the reduction of tetrazolium compound of MTT into purple colored formazan through the mitochondrial reductase enzymes of viable cells. Interestingly, curcumin has the capacity to act through many pathways in cancer cells [40]. The MTT results indicated that the growth of HepG2, MCF7, HT29, and A549 cells was significantly inhibited after the treatment with Cur/ALG-GANPs and curcumin as compared to the untreated cells (*p* ≤ 0.05). The highest inhibition (27.2%, 33.4%, 31.3%, and 32.1%) were found at concentration of 100 µg/mL of Cur/ALG-GANPs for HepG2, MCF7, HT29, and A549 cells, respectively. The effect on four cancer cells was concentration dependent and the concentration of 100 µg/mL was the most remarkable concentration in this regard. For MCF7, HT29 and A549 tested, the similarity in toxicity of both Cur/ALG-GANPs and curcumin was confirmed by the IC_50_ values. It has been concluded that both Cur/ALG-GANPs and curcumin exhibited the higher toxicity toward all tested cell lines after 72 h of exposure.

The efficacy of Cur/ALG-GANPs nanoparticles on liver cancer cells (HepG2) was significantly higher than free curcumin. The IC_50_ values of curcumin loaded nanoparticles in HepG2, HT29, MCF7, and A549 cells were (9.64 ± 1.1), (15.88 ± 1.2), (16.84 ± 1.4), and (16.38 ± 0.3), respectively. It can be suggested that the cytotoxicity of Cur/ALG-GANPs nanoparticles are due to the internalization capacity and enhancement of water solubility. Free curcumin and Cur/ALG-GANPs nanoparticles exhibited a comparable effect on cancer cells which mentions that curcumin retains its anticancer activity even after being encapsulated in ALG-GA NPs nanoparticles.

According to the MTT results, no significant toxicity was recorded for ALG-GA nanoparticles due to the non-toxic effect of polymers toward HepG2, HT29, MCF7, and A549 cell line (Figure 8, Figure 9, Figure 10). As mentioned earlier the coating material had shown some antioxidant properties, which might indicate the interference of curcumin with cell viability in cancer cells by mechanisms other than the antioxidant properties alone.

The higher toxicity of Cur/ALG-GANPs nanoparticles against HepG2 might be attributed to the galactose group of gum arabic which is apparently selective to identify asialoglycoprotein receptor (ASGPR) on the surface of human hepatocytes [41].Various glycoproteins have been involved in cancer and antiviral immunity and can function as adhesives in inflammatory responses with immunological properties.

Therefore, the curcumin cytotoxicity in breast cancer might be related to the chemotherapeutic effect of this compound in drug-resistant breast cancer cells. Previous studies indicated that curcumin growth inhibitory effect in cancer cells might be attributed to its inhibitory effect on NF-KB related pathway which leads to cellular apoptotic response and the modulation of arachidonic acid metabolism [42,43,44].

## 4. Materials and Methods

### 4.1. Materials

Gum Arabic (GA) used in this study was purchased from the ENNASR company (Mw: 2.5 × 10^6^ g. mol^−1^, Khartoum, Sudan). Sodium alginate (ALG: molecular weight = 4.8 × 106 ± 1.8 × 10^5^ Da, nominal viscosity = 300 mPa.s) was bought from Sigma Aldrich (Dteroit, MI, USA). Curcumin (purity, >98%, Mw = 368.36 g/moL, C_12_H_20_O_6_) was bought from Biolution Resources company (Serdang, Selangor, Malaysia). Phosphate buffer solution (PBS) was purchased from R&M (Pekin, China). Fetal Bovine Serum (FBS), Trypsine-EDTA, 3-(4,5-dimethylthiazol-2-yl)-2,5-diphenyltetrazolium bromide (MTT), 1,1-diphenyl-2-picrylhydrazyl (DPPH), Trolox, and Dulbecco’s modified Eagle’s medium (DMEM) high glucose were bought from Sigma Aldrich (St. Loius, MO, USA).

### 4.2. Synthesis of Curcumin Loaded Sodium Alginate-gum Arabic Nanoparticles

The sodium alginate/gum arabic containing curcumin nanoparticles ALG-GANPs were synthesized following ionotropic gelation technique with slight modifications using calcium chloride (CaCl2) as a cross linker [45,46].In the first step gum arabic (0.70g) was dissolved in 50 mL of deionized water. Sodium alginate dispersion was prepared separately by dissolving (0.80g) in 50 mL of deionized water by stirring overnight in a conical flask. Afterwards, the two dispersions were mixed gently under stirring of 1000 rpm for 10 min. An aqueous solution of curcumin was formed in ethanol at a concentration of 1 mg/mL then was added to the dispersion in a ratio of 1:4 and mixed using homogenizer. After exposure to 5 min of ultrasonication for debubbling, the resulting solution was mixed drop-wise to 100 mL of 10% (*w*/*v*) CaCl2 (Calcium chloride) solution for 15min. The curcumin nanoparticles were obtained by freezing the dispersion at −80 °C for 24h then freeze-dried at −50 °C for 72 h. The obtained nanoparticles were then re-dispersed in distilled water and then given a wash followed by centrifugation at 20,000 rpm at 4 °C for 5 min. As a result, nanoparticles were formed and subsequently stored in vials for further characterization.

### 4.3. Characterization of ALG-GA NPs Nanoparticles

#### 4.3.1. ATR-FTIR Spectrometer

Attenuated total reflectance-Fourier transform infrared (ATR-FTIR) spectra of free curcumin, ALG, GA, ALG-GA nanoparticle, and Cur/ALG-GA NPs nanoparticle powder were measured through the range of 400–4000 cm^−1^ and resolution of 4 cm^−1^ on a Perkin-Elmer ATR-FTIR spectrometer (L1600401 Spectrum Two DTGS, Llantrisant, UK).

#### 4.3.2. X-ray Diffraction (XRD)

To assess the crystallinity of free curcumin before and after encapsulation, X-ray diffraction patterns of pure Curcumin, ALG, GA, ALG-GA physical mixture, and Cur/ALG-GA NPs were carried out using Shimadzu refractometer (Tokyo, Japan), and CuK_α_ radiation, 30KV at the range of 2–60°. The scanning rate used was 2° per minute.

#### 4.3.3. Differential Scanning Calorimetry (DSC)

Differential scanning calorimetry analysis was recorded by the use of differential scanning calorimeter (DSC1, STAR System, Mettler Toledo, Zurich, Switzerland) to assess the physical state and investigate the crystallinity of pure curcumin, ALG, GA, ALG-GA physical mixture and Cur/ALG-GA NPs. Heating at a rate of 10 °C/min from 25 to 300 °C under a 50 mL/min nitrogen flow. A blank aluminium crucible was used as a reference for all DSC measurements.

#### 4.3.4. Particle Size and Surface Charge (Zeta Potential) 

The Zeta potential and size of Cur/ALG-GA NPs were characterized using a zeta sizer (Malvern Nano-ZS90, London, UK) with dynamic size at a fixed scattering angle of 90°. The samples were prepared immediately, and the size measurement was done using 0.1 mL of prepared fresh samples with pH 6.7 then diluted in glass cuvette with 0.9 mL of deionized water. For the zeta-potential, the 0.1 mL of samples were diluted in 1.9 mL of deionized water followed by ultrasonic treatment to avoid aggregation and get uniform dispersion then measurement under the 25 °C. All the experiments were carried out in triplicate with a time span of 120 s.

#### 4.3.5. Transmission Electron Microscopy (TEM)

Transmission electron microscopy was used to determine the homogeneity of Cur/ALG-GANPs nanoparticles by the use of (TEM Model CM12 Philips; Eindhoven, Netherlands) operated. A drop of nanoparticle was dropped on the copper grid with 200 mesh size (Ted Pella, Redding, CA, USA) and stained using 0.1% of phosphotungstic acid. The particles were air-dried and measured at an accelerated voltage of 200 KV with a maximum magnification of 50 k times.

### 4.4. Encapsulation Efficiency

The encapsulation efficiency (EE%) of NPs was determined by using ultracentrifugation at 10,000 rpm and 4 °C for 20 min to separate nanoparticles from the medium of non-associated curcumin [47]. After that, nanoparticles were diffused in distilled water and washed under the same condition of centrifugation for 6 min. Furthermore, the collected pellet was dispersed again in 6 mL of distilled water and vortexed well. The dispersion was divided into six Eppendorf tubes, then mixed with 200 µl of methanol, vortexed, and centrifuged. UV-Vis spectrophotometer (Berlin, Germany) at 425 nm was used to quantify the yellowish supernatant and the amount of curcumin was calculated by the use of the following equation:

$$\mathrm{Encapsulation}\;\mathrm{effficency}\;\left(\%\right)=\frac{\mathrm{Total}\;\mathrm{amount}\;\mathrm{of}\;\mathrm{curcumin}\;\mathrm{in}\;\mathrm{nanoparticle}-\mathrm{amount}\;\mathrm{ofcurcumin}\;\mathrm{alone}}{\mathrm{Total}\;\mathrm{amount}\;\mathrm{of}\;\mathrm{curcumin}\;\mathrm{in}\;\mathrm{nanoparticle}}\times 100$$

### 4.5. DPPH scavenging activity of curcumin ALG-GA NPs Nanoparticles

Being stable and free radical, the DPPH has been used in past studies to examine the free radical-scavenging activities of active ingredients. The DPPH scavenging test was performed to evaluate the antioxidant capacity of curcumin nanoparticles [48]. Briefly, 195 µL of DPPH solution (200 µM in methanol) was mixed with 100 µL of each sample solution at a concentration of 50, 75, 100, 125, 150, and 200 µg/mL of Cur/ALG-GANPs and curcumin on a 96-well microplate. The absorbance rate was recorded at 517 nm after shaking for 1 min and kept in darkness for 1 h of incubation. A stock solution of Trolox was prepared (100 µg/mL methanol), and then serially diluted to yield a standard concentration of 50, 75, 100, 125, 150, and 200 µg/mL. A negative control including all reagents except test samples were prepared for this test.

The rate of DPPH scavenging capacity was evaluated by the use of the following equation:

$$\mathrm{DPPH}\;\mathrm{scavenging}\;\mathrm{capacity}\;\left(\%\right)=1-\frac{\left(A_{\mathrm{sample}}-A_{\mathrm{control}}\right)}{A_{\mathrm{blanc}}}\times 100$$

where A sample, A blank, and A sample control were recorded from the absorbance of samples, blank, and positive control. Trolox was used as standard antioxidant.

### 4.6. Cell Culture Study

The cancer cell lines were bought from ATCC (American Type Culture Collection, Manassas, WV, USA) including the human breast cancer (MCF7), human lung cancer (A549), human liver cancer (HepG2), and human colon cancer (HT29) cells. These cells were seeded in DMEM high glucose medium enriched with 10% of Fetal Bovine Serum (FBS) and 1% of penicillin-streptomycin. Cells were then incubated at 37 °C with 5% CO_2_.

### 4.7. Cell Viability Assay (IC_50_)

The 3-(4,5-dimethylthiazol-2-yl)-2,5-diphenyltetrazolium bromide (MTT) assay was used to measure the efficacy of curcumin nanoparticles on HT29, MCF7, A549, and HepG2 cells.

The cells were plated by adding 200 µL of 1 × 10^5^ cells/mL of cancer cell suspensions into 96-well plate then incubated at 37 °C and 5% CO_2_ for 24 h. The cells were then treated with (Cur) and ALG-GA NPs of different concentrations (1.56–100 µg/mL) and chemotherapeutic controls at 0.156 to 10 µg/mL (doxorubicin for MCF7, 5-Fluorouracil for HT29, Cisplatin for A549 and Tamoxifen for HepG2 cells). After 24, 48 and 72h of incubation, twenty microliters of 5 mg/mL of MTT was incubated in each well for 4 h. DMSO (100 µL) was added to each well to solubilize blue formazan precipitate. Afterwards, the absorbance was recorded at 570 nm using by the use of microplate reader. The IC_50_ values were calculated from the concentrations of curcumin and ALG-GA NPs that inhibits 50% of cell growth. The measurements were carried out in triplicate.

### 4.8. Statistical Analysis

The measurements were conducted in triplicate. The data were presented as mean ± SD. All analysis of variance was performed based on ANOVA with the value of *p* < 0.05 to be of statistical significance using SPSS software (IBM SPSS Office, Chicago, IL, USA).

## 5. Conclusions

This study signifies the successful nanoencapsulation of curcumin into sodium alginate-gum arabic nanoparticles by using the ionotropic gelation technique. The results suggested that the characterization of Cur/ALG-GANPs nanoparticles using ATR-FTIR, XRD, DSC, and TEM confirm the incorporation of curcumin into ALG-GA nanocavity. Taking into accounts, the results provide information about the encapsulation of curcumin into ALG-GA nanoparticles which can lead to important evaluations of the physicochemical properties of Cur/ALG-GANPs such as stability and morphology. It is confirmed that Cur/ALG-GA led to much more toxicity effects toward HepG2, MCF7, HT29 and A549 cells than the free curcumin with more potent toxicity against HepG2 cells. The highest inhibition activity of 27.2%, 33.4%, 31.3%, and 32.1% against HepG2, MCF7, HT29, and A549 cells, respectively, were found at the concentration of 100 µg/mL of Cur/ALG-GANPs. Based on the outcomes retrieved from the MTT cytotoxicity assay, it can be concluded that the Cur/ALG-GANPs nanoparticles have a therapeutic potential in the prevention and treatment of solid malignancies, such as hepatic, breast, cervical, and skin cancer. These preliminary findings provide the baseline for future approaches towards the discovery of therapeutic mechanisms in vitro and in vivo for the improvement of drug delivery systems.

## Figures and Tables

**Table 1:** Size, PDI, and Zeta potential of Cur/ALG-GANPs.

Size distribution	Zeta potential	PDI
From 10 ± 0.3 to 190 ± 0.11	−15 mV ± 0.22	0.35 ± 0.17

**Table 2:** The IC_50_ values for HepG2, MCF7, A549 and HT29 cells generated from MTT assay following exposure to Cur and Cur/ALG-GANPs for 24, 48 and 72 h.

Cell Line	Duration (h)	Cytotoxicity Assay	
		IC_50_ (Cur)	IC_50_ (Cur/ALG-GANPs)
HepG2	24	48.70 ± 0.33	36.99 ± 0.87
	48	43.73 ± 0.26	24.62 ± 0.93
	72	19.02 ± 1.3	9.64 ± 1.1
MCF7	24	68.20 ± 0.73	48.40 ± 2.13
	48	55.86 ± 0.26	33.26 ± 0.96
	72	32.1 ± 1.3	16.84 ± 1.4
A549	24	69.1 ± 0.64	41.16 ± 0.77
	48	48.16 ± 1.3	34.75 ± 0.64
	72	29.17 ± 0.4	16.38 ± 0.3
HT29	24	78.04 ± 1.22	49.06 ± 1.26
	48	48.28 ± 1.75	32.82 ± 2.31
	72	28.02 ± 1.1	15.88 ± 1.2

**Table 3:** IC_50_ of Tamoxifen, Doxorubicin, 5-Fluorouracil and Cisplatin on HepG2, MCF7, A549 and HT29 after 24, 48 and 72 h of incubation.

Cell Lines	Duration (hours)	IC_50_
HepG2		IC_50_ (Tamoxifen)
	24	4.94 ± 1.16
	48	3.80 ± 0.66
	72	2.1 ± 0.9
MCF7		IC_50_ (Doxorubicin)
	24	2.46 ± 0.34
	48	1.93 ± 1.13
	72	0.9 ± 0.78
HT29		IC_50_ (5-Fluorouracil)
	24	3.26 ± 0.27
	48	2.57 ± 1.17
	72	1.27 ± 0.8
A549		IC_50_ (Cisplatin)
	24	2.47 ± 0.67
	48	1.95 ± 0.81
	72	1.04 ± 0.4
